# Digital volume correlation analysis of polylactic acid based fused filament fabrication printed composites

**DOI:** 10.1177/00219983211020500

**Published:** 2021-05-27

**Authors:** Cristofaro S Timpano, Garrett W Melenka

**Affiliations:** Department of Mechanical Engineering, Lassonde School of Engineering, Canada

**Keywords:** Additive manufacturing, fused filament fabrication, micro-computed tomography, digital volume correlation, composites

## Abstract

Fused filament fabrication (FFF) has rapidly begun to see implementation in industrial fields as a method of rapid manufacturing. Traditional FFF parts are made from a single thermoplastic polymer. The polymer is heated to its melting point and deposited on a work bed where a model is gradually built from the base up. While traditional FFF parts have low mechanical properties, a reinforcing phase allows for improved mechanical properties. The addition of a reinforcing material to the base polymer and complex internal microstructure of the 3 D printed party leads to anisotropic mechanical properties. Thus, these materials’ mechanical properties become challenging to characterize using traditional measurement techniques due to the previously mentioned factors. Therefore, it is essential to develop a method in which mechanical properties can be measured and analyzed. This study aims to characterize the mechanical behaviour under a uniaxial tensile load of an FFF produced polylactic acid (PLA)-copper particulate composite. The internal response of the FFF sample was imaged using micro-computed tomography at predetermined loads. The μ-CT images were input into an open-source digital volume correlation (DVC) software to measure the internal displacements and strain tensor fields. The study results show the development of different strain fields and interior features of the FFF parts.

## Introduction

The emergence of additive manufacturing (AM) has provided a powerful tool for the rapid design and fabrication of parts for engineering applications. Fused filament fabrication (FFF) has become a popular option in the industrial sector due to the wide selection of compatible thermoplastic polymers with the process.^1^ Additionally, the process is well suited for implementation into current manufacturing operations for mass production. Traditional FFF parts are held back due to their lower mechanical properties and inconsistent quality. The poor material strength of traditional FFF parts results in them typically being only suitable for prototyping.^2^ Thus, to improve the material strength and improve AM produced pieces, researchers have begun to produce AM components composed of multiple materials. To this end, researchers have studied the effects of the addition of various reinforcing materials such as carbon nanotubes, graphene, or metal particles to the base polymer.^1,3^ These 3 D printed composite materials help address the lack of strength of traditional AM and FFF produced parts but further complicate the already complex material analysis.

Before discussing the challenges involved in composite FFF parts, it is crucial first to discuss the issues with analyzing single-phase FFF parts. FFF parts exhibit anisotropic mechanical properties due to their complex microstructure. Regions of high localized porosity and imperfect layer bonding contribute to FFF parts’ complex microstructure and anisotropic material properties.^4,5^ This is further complicated by adding a reinforcing phase where factors such as particle loading, size, and adhesion affect the part's mechanical properties.^6^ Traditional testing methods of material properties, such as strain gauges or extensometers, are unreliable for materials produced through FFF. Strain gauges and extensometer are inherently discreet surface level measurement system. Thus, the anisotropic material properties and heterogenous deformation field cannot be captured. Additionally, the build parameter of FFF parts intrinsically influnce the mechanical properties, by way of altering the microstructure of the material. Macroscopic meaurements, like strain gauges, are not able to investigate strain within the internal microstructure of a component.^7^

Thus, researchers have proposed an alternative approach for analyzing samples with anisotropic material properties and heterogenous deformation fields known as Digital Image Correlation (DIC).^8^ DIC captures images during material testing from either a 2 D or 3 D stereo camera set up to visualize the material’s deformation.^9^ These images are then utilized as input for a software algorithm to calculate deformation and strain.^9^ DIC provides an advantage over traditional testing methods as a full-field profile of deformation and strain can be determined.

Though DIC offers many advantages, its adoption for material analysis for FFF produced parts has been limited. Zaldivir *et al.* were one of the first groups to use DIC to study the effects of build orientation on FFF fabricated ULTEM 9085 tensile coupons.^5^ The study showed that deformation developed in a highly anisotropic manner. Additionally, the build orientation had a significant effect on the material strength and formation of strain irregularities within the sample. Goodarzi Hosseinabadi *et al.* provided a comparison study aided by DIC on Acrylonitrile butadiene styrene (ABS) FFF honeycomb parts and photocured polyjet honeycomb parts.^10^ The usage of DIC allowed for the resolution of strain fields for samples under a compressive load. An extension of this study was completed to understand the shearing effects of a compressive load on the piece. This study showed that shear occurred in three stages, which agreed with finite element analysis (FEA) models.^11^

While DIC allows for full field testing of FFF parts, the cameras used for capturing images are limited to only surface level results.^12^ Thus, it is difficult to capture and relate strain results throughout the entire 3 D microstructure. Internal strain measurement in FFF components is crucial for materials that are highly influenced by there microstructure via the DIC technique. The advent of micro-computed tomography (µ-CT) has provided a non-destructive method to capture the interior features of materials on the sub-millimeter level. µ-CT operates by passing x-rays through a rotating sample to obtain a volumetric set of images.^12^ As the x-rays pass through the material, they are attenuated based on the density of the material they pass through and collected by an x-ray receptor.^12^ This data is used to form shadow-projections of the images, which are reconstructed into 2 D cross-sections of the volume.^13^

µ-CT has recently emerged as a valuable tool for the analysis of AM produced parts. Researchers have begun to utilize µ-CT to image the microstructure, quality, and printing parameters of various AM parts. The effects of printing parameters such as temperature, raster orientation, and extrusion width on the materials’ microstructures are of particular interest.^14–17^ Through the aid of the µ-CT images, accurate measurements can be made on critical structural parameters such as porosity and raster geometry. This paper proposes to utilize µ-CT to measure changes in the microstructure of FFF composites to external loading.

With the 3 D data set provided through µ-CT, the ability to capture volumetric full-field strain data is made possible via digital volume correlation (DVC). As a 3 D extension of DIC, DVC was first outlined in the ground-breaking paper by Bay et al.^18^ DVC operates by discretizing a reference and displaced data set into a series of subsets. For an accurate correlation, these subsets must contain features with a random variation of greyscale values. Greyscale variation can be formed naturally from the natural microstructure or as a result of elements externally seeded into the material. Features in each subset are tracked using an objective function to determine the displacement from the reference position to the deformed position. This data can then be directly converted into strain, thus providing a volumetric model of the internal deformation.

DVC is an emerging method for displacement and strain measurement; therefore, current literature in these areas is limited. DVC was first used in bioscience, particularly for obtaining existing strain fields in trabecular bone samples.^19–21^ Current DVC research has begun to extend into engineering material science. DVC has proved a powerful tool for analyzing heterogeneous materials such as wood, metal alloys, and composites laminates.^22–29^ However, the DVC technique has been sparsely utilized to investigate the mechanical properties in additive manufacturing.^4^

This study aims to address the current gaps present in the literature on the analysis and application of µ-CT and DVC for FFF produced composite materials. This work addresses the significant challenge for capturing full volumetric strain measurement within the microstructure of FFF materials. Current surface measurement methods like strain gauges or DIC are incapable of studying the interaction between FFF materials’ microstructure and mechanical properties. µ-CT and DVC provide a method for capturing FFF parts’ microstructure and its deformation during loading, which can be directly converted to strain results. This paper provides a fundamental first step towards capturing FFF parts' deformation behaviour, which can be built upon in future works that aim to provide a complete analysis of build parameter effects on the deformation mechanics of 3 D printed parts and their composites.

Mainly, this work demonstrates the viability and advantages of utilizing µ-CT and DVC to obtain structural and mechanical data. For this purpose, an FFF part was constructed according to a modified ASTM-D648 14 Type V compatible with the utilized μ-CT integrated material test stage (MTS). The samples were then imaged at each stage of a 3-part stepwise load to capture deformation progression on the sample’s microstructure. The µ-CT images were analyzed to demonstrate the bulk response of the material’s microstructure, porosity, and air gap orientation. This information was then utilized to lead the discussion on DVC results for internal displacement and strain fields.

The resulting µ-CT tomographs were utilized as an input for DVC to capture the internal displacement and strain fields. To this end, an open-source DVC software known as Fast Iterative Digital Volume Correlation (FIDVC) was employed to calculate the 3 D strain and displacement.^30^ FIDVC operates based on an iterative algorithm, where the correlation window size becomes smaller through each pass to improve resolution on the sample’s displacement. In this study, a 100 N load was first applied to the test sample as a reference point, with subsequent loads applied at 150 N and 200 N load states. Due to the coupon’s deformation experience and reduced computational burden, it was necessary to reduce the dataset to a quarter of their original resolution. Measurements of the longitudinal displacements and longitudinal and transverse strains were then calculated via DVC at the 150 N and 200 N load conditions by utilizing the 100 N load as a reference point. Renderings of the displacement and strain behaviour were then made, allowing for the 3 D visualization and analysis of the sample’s mechanical response to the uniaxial load condition. The volumetric displacement and strain results can be built upon in later works allowing for input or direct comparison to improve current FEA models.^31–34^

## Methodology

### Manufacturing

**Figure 1.**
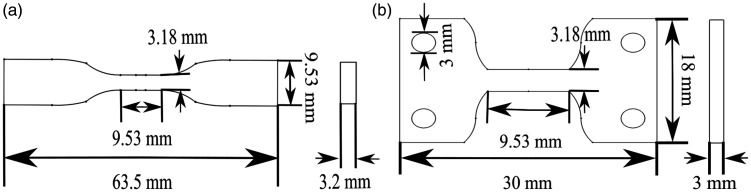
Cross-section sketch of the tensile couple: (a) ASTM D638-14 type V (b) modified design to fit in MTS.

Samples were 3 D printing using a 1.75 mm diameter PLA filament with embedded copper particles (Metal Filled PLA, CCTree-Mech solutions LTD, Concord, Ontario). Samples were designed and printed into tensile coupons based on ASTM D638-14 Type V.^35^ Due to the MTS’s restrictive size (440 N integrated test stage, Bruker, Belgium), modifications were required to the geometry. The coupon’s total length was reduced to 30 mm and thickness to 3 mm while maintaining the gauge length and width to ensure the coupon fit within the MTS’s. Additionally, the end tabs were increased to a width of 18 mm, and a pair of 3 mm holes were included to improve compatibility with the MTS. The difference between the ASTM D638-14 type V tensile coupon and the modified used in the current study are seen in Figure 1(a) and (b), respectively.

**Figure 2.**
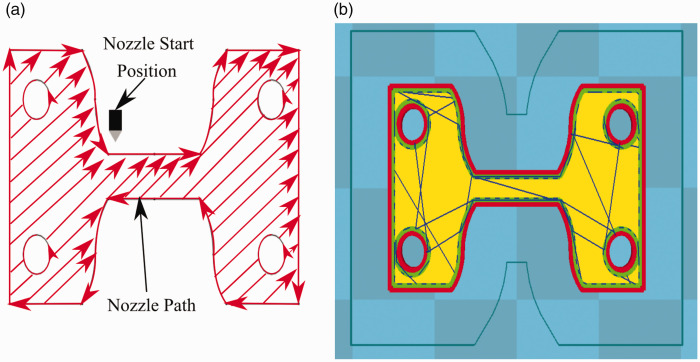
Representation of the tensile coupons printing scheme: (a) nozzle path, (b) slicer view of the center layer of coupon.

**Figure 3.**
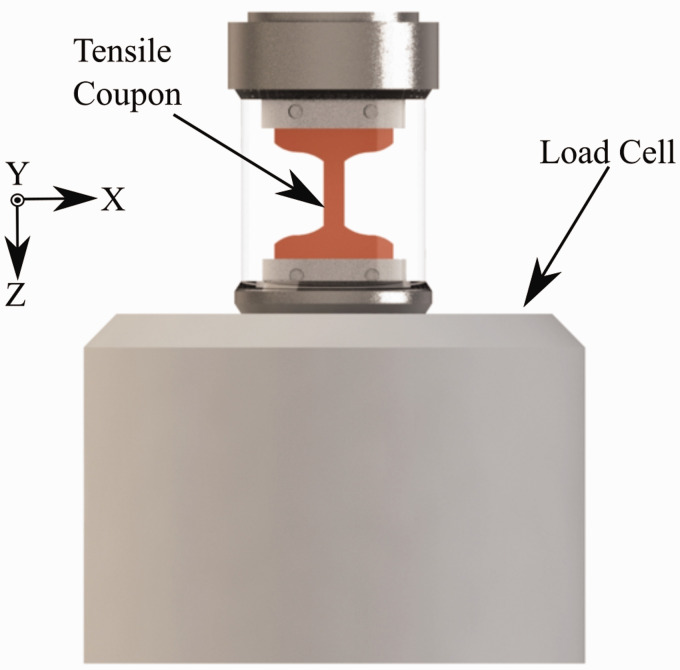
Model render of the MTS loaded with tensile coupon.

Modelling the tensile coupons was done using a computer-aided design (CAD) modelling package (Solidworks, S2018, Dassault Systemes, Vélizy-Villacoublay, France). The sample CAD model was sliced using open-source software (Cura, 15.04.06, Ultimaker, Geldermalsen, Netherlands). The sliced sample was finally printed using a desktop 3 D printer (Select mini V2, Monoprice, Rancho Cucamonga, California, United States) fitted with a 0.4 mm nozzle diameter. Table 1 shows a list of the printing parameters utilized to manufacture the tensile coupons. Samples were printed with a 100% infill at a layer thickness of 0.10 mm. The nozzle’s temperature was set to 210°C, and the build plate temperature was 70°C. Samples were printed first by creating a 0.8 mm shell on each layer that outlined the coupon’s geometry before completing the infill with rasters on a +45°/−45°. In Figure 2(a), the nozzle start position and path for a single layer is presented. Figure 2(b) shows the center layer’s corresponding view as produced by the utilized slicer software. To improve layer adhesion to the printer bed was increased by using the “raft” setting in Cura. A render of the tensile coupon housed inside the MTS is shown in Figure 3. The tensile coupon is connected to the MTS with top and bottom clamps. Pressure is applied to the sample through the clamps through by a pair of screws which pass through the test coupon. Surrounding the tensile coupon is a radiolucent pane, which provides some rigidity to the test frame during loading. During loading, the top end of the coupon remains static as the bottom face is displaced downward.

**Table 1.** Manufacturing parameters for slicer software utilized to produced tensile coupon.

Manufacturing parameter	Parameter value
Layer Thickness	0.1 mm
Infill Density	100 %
Fill Direction	+45°/−45°
Shell Thickness	0.8 mm
Nozzle Temperature	210°C
Bed Temperature	70°C

### Micro-computed tomography

**Figure 4.**
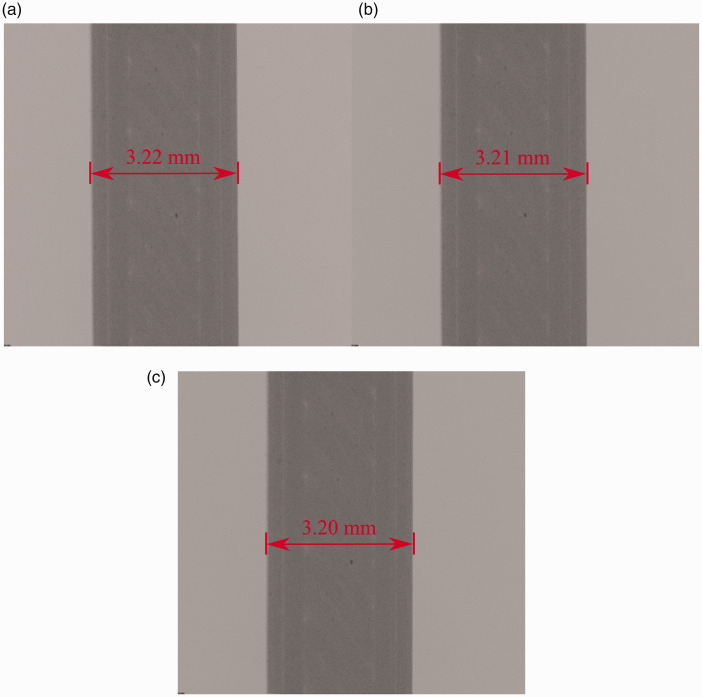
Shadow projections of the copper PLA sample for (a) 100 N, (b) 150 N, and (c) 200 N loads.

The copper PLA tensile coupons were imaged using a desktop µ-CT (Skyscan, 1272 µ-CT scanner, Bruker, Belgium.). Table 2 presents a complete list of the µ-CT inputs employed to image the sample. The PLA samples were imaged using a pixel size of 1.6 µm/px with a 4904x3280 pixel resolution. Settings were selected to ensure the embedded copper particles were captured in the image while allowing for a majority of the gauge length to be captured. The source voltage and current used in this study was 65 kV and 125 μA. Due to metals’ tendency to cause metal scattering artifacts and beam hardening, a 0.5 mm aluminum filter was used to reduce possible metal artifacts that may appear in tomographs post-reconstruction. Three scans were completed successively at loads of 100 N, 150 N, and 200 with a 0.2° rotation step. The final dataset size before reconstruction for each load contained 938 shadow projections. A single shadow projection from each dataset is shown in Figure 4. Measurements of the sample thickness show a decrease in the width of approximately 0.01 mm per load step. This decrease in sample width as the tensile load increases is indicative of a reduction of cross-section size. Of note, the measurements shown are larger than the 3.18 mm width of the solid model used to manufacture the sample. The larger width seen in the sample shadow projections indicates manufacturing inconsistencies on the part of the 3 D printer. The increase in the model’s width is shown to be between 0.02 mm to 0.04 mm, which is well within the typical tolerancing of the FFF printed part.^36^

**Table 2.** List of scan parameters utilized to image the tensile coupon.

µ-CT Parameter	Setting
Source Voltage	65 kV
Source Current	125 µA
Resolution (WxH)	4904 × 3280 px
Pixel Size	1.6 µm/px
Filter	0.5 mm Aluminum
Rotation Step	0.2°

### Reconstruction

A μ-CT reconstruction package (NRecon version 1.7.1.0, Bruker, Belgium) was utilized to convert the shadow projection into a series of perpendicular cross-sectional images. Before reconstruction, a range of greyscale values must be selected to provide image contrast and allow for the visualization of microstructure features. It is recommended by the software package provider to set the lower limit of the attenuation range to 0 (−1000 HU) and the upper value to be around 10–20% greater than the maximum variation in greyscale.^37^ Using the criteria previously presented, the attenuation range selection was 0 (−1000 HU) to 0.5 (31,768 HU) for the 100 N and 200 N and 0 (−1000 HU) to 0.25 (15,384 HU) for the 150 N load. It is important to note that the attenuation ranger utilized for the 150 N load differs from the 100 N and 200 N load. The reason being is that samples were scan using a 180° scan rotation. Thus, starting position for the scans differed between the 150 N sample and the 100 and 200 N loads. Therefore, it was necessary to set the attenuation range differently to meet the previously mentioned criteria.

**Figure 5.**
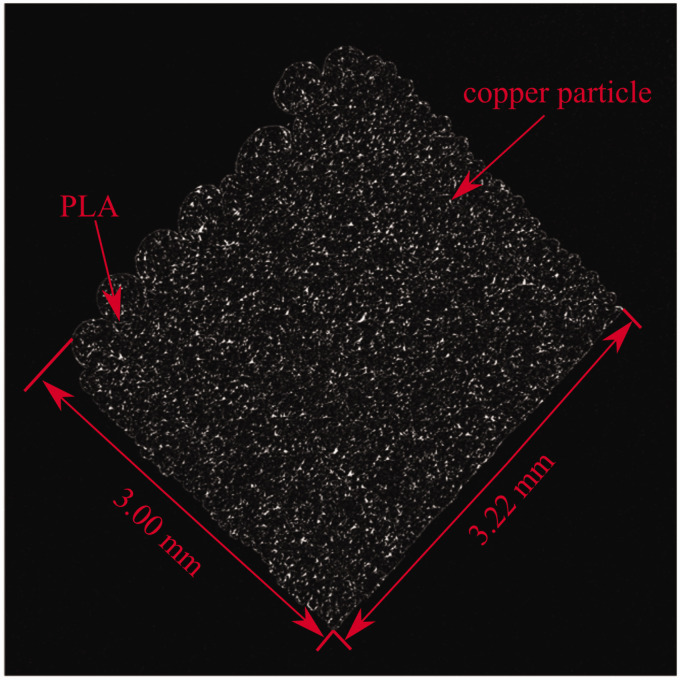
Reconstructed cross-section of copper PLA sample with enhanced contrast.

While procedures were put in place to reduce noise and artifacts before scanning, this does not eliminate the possibility that artifacts appear. Thus, a 49% beam hardening filter and a 10% ring artifact filter was applied to the image set during reconstruction. The beam hardening filter’s aggressive use compensated for the streaking caused by metal particles’ presence within the sample.^38^ Figure 5 shows an example of a reconstructed cross-section from the 100 N dataset. The contrast in Figure 5 was enhanced beyond what was used in the current study to provide better microstructure visibility. Within the tomographs, the copper particles can be easily distinguished from the PLA due to higher greyscale values resulting from their higher density.

### Image segmentation

#### Image quality assessment

Before performing DVC measurements, the quality of the tomographic images must be assessed for DVC compatibility. For DVC measurements, the greyscale distribution and particle quantification can be used to evaluate image data quality. These parameters were measured within a single correlation window, which was cropped out of the full resolution 100 N dataset equal to 128 x 128 x 128 voxels or 0.2048 x 0.2048 x 0.2048 mm. The greyscale histogram of the 3 D image stack was then measured using MATLAB (MATLAB R2018a, The MathWorks, Natick, Mass) digital image processing toolbox. The particle numbers’ quantification was done through a commercial CT image segmentation software (CTan version 1.16.8.0, Bruker μCT, Belgium). As a requirement to quantify features within the images, it is first required that the dataset be binarized. Binarization was done by thresholding the dataset between greyscale values of 11–255, which highlights the internal particles. Particles were then measured using the “3 D analysis” command, which counts all-white features within the image sequence and measures their area and volume.

#### Cross-section analysis

Measurements of sample manufacturing variability and the microstructure response to the tensile load were first measured through an image segmentation procedure before a more detailed analysis of the latter was conducted utilizing DVC. Of particular interest for this paper was quantifying the change of cross-sectional area and air gaps between rasters with the tensile loading. This measurement was performed via CT image segmentation software. Typically, segmentation in CTAn is broken into three steps: 1) binarization, 2) filtering, and 3) morphological operation. For quantifying the sample’s cross-sectional area, the images were segmented to show one single-phase cross-section. Due to the large sizes of the reconstructed data, a 3 mm section of each sample was examined to decrease the computational burden.

**Figure 6.**
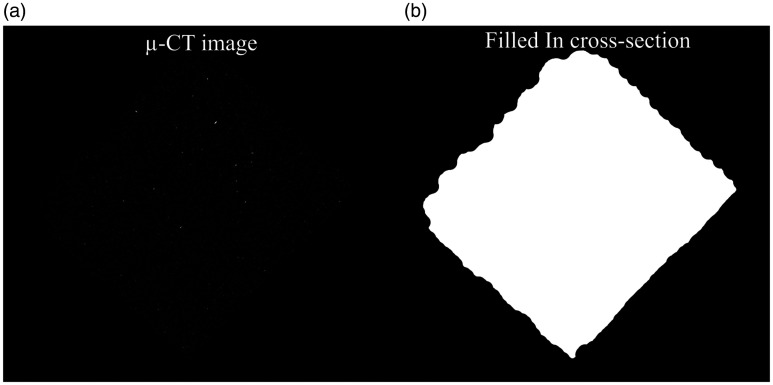
Segmentation of copper PLA sample cross-section: (a) µ-CT image, and (b) filled sample cross-section.

As a result of the different attenuation ranges used to reconstruct the data, the 100 and 200 N dataset and 150 N dataset were processed slightly differently. The steps utilized for these two procedures are listed in Tables 3 and 4 respectively. The 100 N and 200 N datasets were binarized by thresholding the sample between greyscale values of 10–255. This binarization was shown to provide the best compromise of realizing the sample’s internal features and reduce noise. Filtration of the resultant noise from binarization was done by performing a despeckling operation in 2 D space, eliminating all white details below a size of 8 pixels. To fill the material’s internal structure, a morphological closing operation that consists of a dilation followed by erosion was used with a 10 pixel round kernel. While the morphological closing did homogenize most of the internal cross-section, it also increased the size of some of the noise not eliminated by the despeckling operation. Thus, an erosion operation of 5 pixels was utilized to eliminate this noise, followed by a 5-pixel dilation to address a subsequent reduction in size resulting from the erosion. As a final step to close a few outstanding gaps in the cross-section, a 60-pixel square kernel morphological close was completed. The effects of this procedure on the µ-CT images are shown in Figure 6. Figure 6(a) highlight the µ-CT tomograph before image segmentation. Note that this has been intentionally left dark to highlight the true greyscale variation. While in Figure 6(b) the realized cross-sectional from the µ-CT image segmentation procedure is shown.

**Table 3.** Image processing parameters used to define the cross-sectional area of 100 N and 200 N datasets.

Image processing operation	Image processing parameter
Binarization (Greyscale threshold)	10–255
2 D Despeckling (white pixels)	<8 pixels
2 D Morphological Closing (Round)	10 pixels
2 D Erosion (square)	5 pixels
2 D Dilatation (square)	5 pixels
2 D Morphological Closing (Square)	60 pixels

**Table 4.** Image processing parameters used to define the cross-sectional area of 150 N datasets.

Image processing operation	Image processing parameter
Binarization (Greyscale threshold)	23–255
2 D Despeckling (white pixels)	<15 pixels
2 D Morphological Closing (Round)	10 pixels
2 D Erosion (square)	5 pixels
2 D Dilatation (square)	5 pixels
2 D Morphological Closing (Square)	60 pixels

The procedure used to process the 150 N dataset followed the same general steps presented in Table 3. However, due to the difference in the attenuation range used for reconstructing the 150 N dataset, the input parameters differed slightly from the 100 N and 200 N datasets. In practice, the differing attenuation range only affects the binarization and despeckling steps. Due to the narrower attenuation range, the greyscale thresholding utilized to binarize the image was set between 23-255. As a consequence of this, the noise surrounding the sample was much more extensive. Thus, a more aggressively sized despeckling operation was performed by removing white features below a size of 15 pixels. Beyond this step, the remaining procedures and reasoning for utilization are the same for the 100 N and 200 N dataset. The entire utilized process is outlined in Table 4.

After segmenting the images, the cross-sectional area of each image within the stack was quantified. This quantification was done by utilizing the image processing toolbox in MATLAB. A series of statistical measurements can be made within MATLAB on the binarized image using the ‘regionprops’ command. For this study, the area parameter was used, which measures the size of all connected white pixels within a single image. By inputting each segmented image individually through the software, the area and its variation along the 3 mm section were computed.

#### Air gap measurements

**Figure 7.**
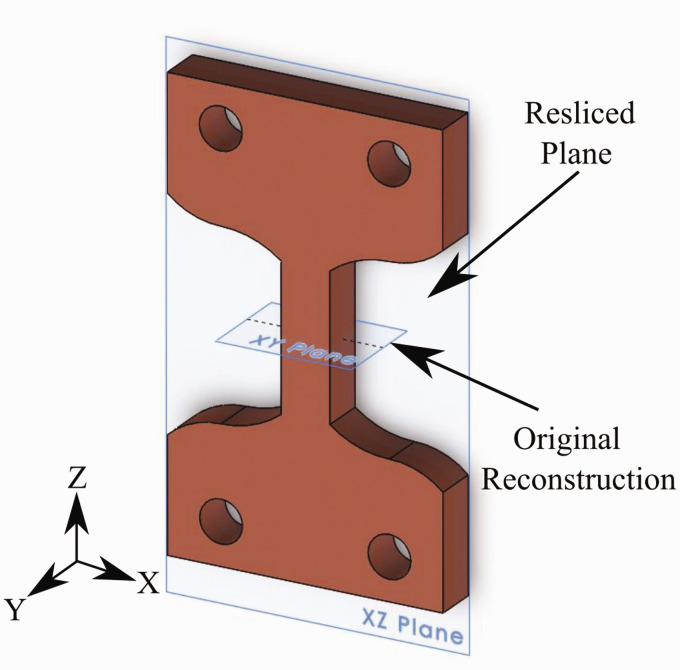
Schematic showing the original reconstruction plane from micro-CT, and resliced plane.

A similar image segmentation procedure was utilized to quantify the air gaps present in the sample. Quantification of the air gaps allows for assessing the actual sample parameters post-manufacturing and how they change during the material’s mechanical testing. Additionally, this will enable the examination and relation of the strain fields obtained from the DVC analysis to printing patterns. To perform this measurement, it was useful to reslice the reconstructed data in the layer thickness direction to improve visualization. Each dataset’s reslicing was achieved by utilizing an open-source java-based image processing program (Fiji, National Institutes of Health, Bethesda, MD). As seen in Figure 5, the reconstructed data is slightly rotated; thus, before reslicing the data, images were first rotated 45° using a bilinear approximation interpolation scheme to realign the sample. An image stack could then obtain in the thickness direction by utilizing the reslice command. Figure 7 provides a comparison of the reconstructed images and the reconstructed dataset directions. The original reconstructed images are shown to lay in the XY plane of the sample and vary along the Z direction. By reslicing the dataset into the XZ plane, the cross-section area’s variation along Y or the nozzle’s height-wise direction, visualization of the airgaps becomes possible.

After reslicing the data, a segmentation procedure was again utilized to visualize and measure the sample air gaps. Unlike the cross-sectional analysis, each dataset was processed using the same parameters as it was found the best range for binarization was to contain the entire greyscale range. A similar image segmentation procedure was followed to that used in a previous µ-CT study to quantify open pores in PVDF foams.^39^ For this analysis, the datasets were reduced to half their original resolution to decrease computation time.

**Figure 8.**
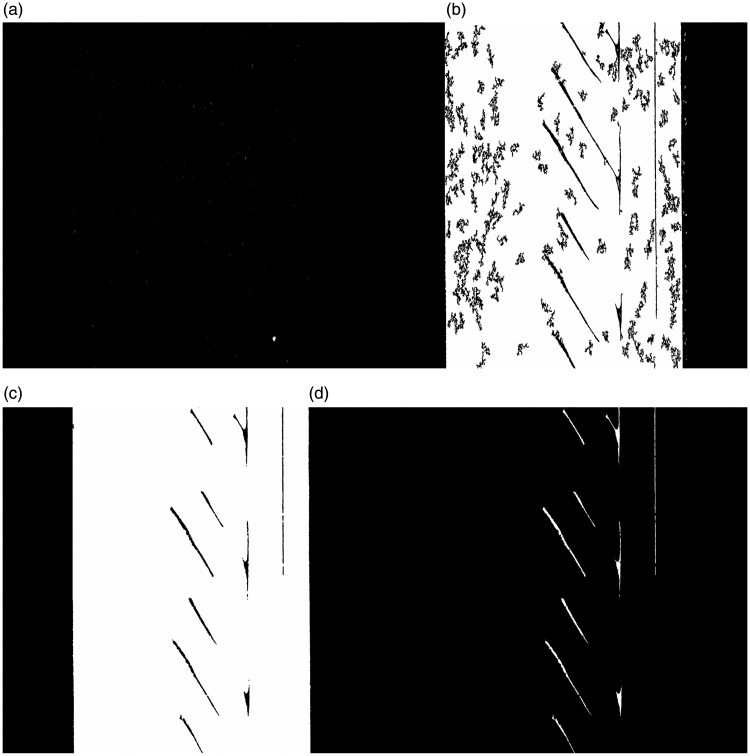
Pores identification steps for copper PLA sample: (a) µ-CT image, (b) filtered and despeckled, (c) large despeckled and closed (d) inversed image highlighting air gaps.

The image processing parameters for each dataset are shown in Table 5. As a first step toward filling in the sample, small black features that did not constitute air gaps were removed. This was done in two stages first, by removing all-black features surrounded by white with a volume of 50 voxels and second, by eliminating all features with an area of 200 pixels or less. After removing the majority of small black features within the sample, three items still existed within the image data: the significant air gaps, white noise, and unfilled gaps in the PLA that do not constitute air gaps. A 2 D white despeckling operation was undergone to remove all white pixels below 200 pixels to remove the image’s white noise. Removal of the unfilled openings in PLA, a 3 D despeckling was done to remove voxels below the size of 2000 voxels. A small morphological close followed this procedure in 3 D space to reduce the size of any remaining gaps before passing over the data one final time with the same 2000 voxel despeckling operation. After these steps, the PLA was successfully realized as white pixels within the image. Thus, to analyze the air gaps, the images were inverted by subtracting the original image from a region of interest (ROI) surrounding the sample. Finally, measurements were conducted on these images using the built-in 3 D analysis tool to quantify the volume of the air gaps. The resulting images for each significant step in the image segmentation process are shown in Figure 8. Figure 8(a) was notably left dark to reflect the true contrast of the image before binarization and segmentation.

**Table 5.** Image processing parameters to segment out sample air gaps for 100 N, 150 N, and 200 N dataset.

Image processing operation	Image processing parameter
Binarization (Greyscale Threshold)	1–255
Despeckling (3 D black voxels)	<50 voxels
Despeckling (2 D black pixels)	<300 pixels
Despeckling (2 D white pixels)	<200 pixels
Despeckling (3 D black voxels)	<2000 voxels
Morphological Close (3 D Round)	1 Voxel
Despeckling (3 D black voxels)	<2000 voxels
Arithmetic Operation	Sub ROI

### Digital Volume correlation

#### Fundamental principles of FIDVC

**Figure 9.**
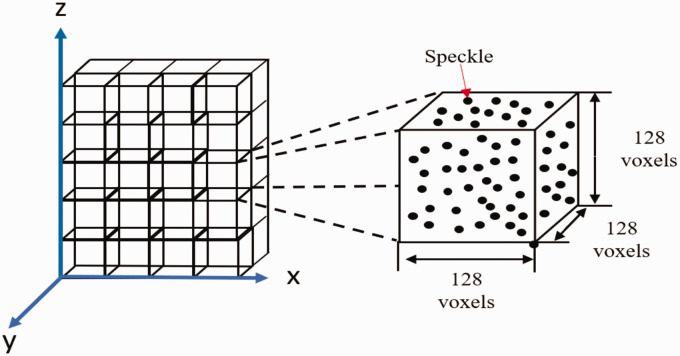
Illustration of the DVC discretization process of the sample into the individual speckled subsets.

Calculations of internal displacements and strains were determined using an open-source MATLAB based DVC software (FIDVC, version 1.2.4).^30^ The DVC software utilized operates by first discretizing a volume into a series of subsets based on a user-selected subset size and subset spacing. The DVC software used an iterative approach where the subset’s size is reduced between passes to a volume as small as 32 × 32 × 32 voxel to increase correlation accuracy. After correlation, displacement is provided by four separate 3 D arrays. These four arrays consisted of displacements in the x-direction, y-direction, z-direction, and displacement magnitude. Figure 9 shows a representation of the dataset after discretization. Each subset in the array contains a single value representing the estimated displacement in that region. Not shown in Figure 9 is the overlap of each subset. After discretization, each subset is overlapped by a specified percentage from the user with all other surrounding subsets to improve spatial resolution.

Strain measurements were computed using an open-source extension to the DVC software (LD-3D-TFM, version 1.1). This extension calculates strain values directly from the displacement data according to the Lagrangian linear elastic strain formulation. The Lagrangian linear elastic strain formulation was chosen due to the brittle nature of the FFF sample and low load applied to result in the sample remaining in this strain region before fracture.^40^ Equation (1) shows the general form for Lagrangian linear elastic strain tensor where *E* represents the strain tensor, *i* represents the first coordinate direction, *j* the second coordinate direction, u the displacement, and *x* the direction of the displacement.^41^ Equation (2) shows the final strain tensor matrix with six unique entries: the three normal strain (*ε*) and three shear strain (τ). Each strain tensor component is made up of a 3 D matrix of equal size to the displacement matrix. Custom code was used to convert the matrices into the Visualization Toolkit (VTK) to visualize the data in 3 D.^42^ Visualization and analysis were then carried out using an open-source scientific visualization platform (Paraview, 5.8.0, Sandia National Laboratories, Kitware Inc, Los Alamos National Laboratory).

(1.1) $$E_{ij}=\frac{1}{2}\left(\frac{\partial u_{i}}{\partial x_{j}}+\frac{\partial u_{j}}{\partial x_{i}}\right)i=1\text{,}2,3\;j=1\text{,}2,3$$

(1.2) $$\;\varepsilon =\left[\begin{array}{ccc} \varepsilon_{xx} & \tau_{xy} & \tau_{xz}\\ \tau_{yx} & \varepsilon_{yy} & \tau_{yz}\\ \tau_{zx} & \tau_{zy} & \varepsilon_{zz} \end{array}\right]$$

#### Digital volume correlation input parameters

This study’s DVC measurement was conduction on a server computer (Precision T5600, Dell, Round Rock, Texas) equipped with 112 Gb of Ram and two Intel® Xeon® CPU E5-2680 2701 Mhz 8 core processors. Before the DVC was run first, the dataset was downsampled to a quarter of its original size. The downsampling algorithm works by averaging voxel greyscale level in a cube with dimensions equal to the user-selected resizing. For this study, a 4 × 4 × 4 downsampling voxel was utilized. While downsampling the dataset reduces image resolution and may introduce a source of error, this process provided many benefits that aided in analysing displacement and strain. The first benefit associated with downsampling is the associated reduction computational burden required to perform DVC on the full volume of interest (VOI). Additionally, this can increase the size of data contain within a correlation window. With the restrictive number of subset sizes provide, for the full resolution, the data was restrained to between a 128 × 128 × 128 voxel (0.2048 × 0.2048 × 0.2048 mm) subset size and 32 × 32 × 32 voxel (0.052 × 0.052 × 0.052 mm) subset size. Considering the rigidity of copper filled PLA sample, which has previously been presented by Liu *et al.*, the displacements seen are likely to exceed some of the sizes of these subsets.^43^ Thus, by reducing the data, it was found that enough detail is provided within each subset so that the DVC software will be effectively able to capture the sample displacement and strain caused by the applied forces of 100 N, 150 N, and 200 N.

Table 6 shows a list of input parameters utilized for the DVC studies. The preload state of 100 N was used as the reference dataset from which displacements and strains were calculated. A list of the program input parameters can be seen in Table 6. The analysis was started with a course subset size of 128 × 128 × 128 pixels, and over four iterations, the correlation subsets were reduced to 32 × 32 × 32 pixels. A subset overlap was of 50% was utilized, which has previously been shown to provide the most accurate results for DVC measurements.^44^ Finally, the mesh spacing was set to a size of 16, which is equivalent to half the size of the final subset as recommended by the software providers.^30^

**Table 6:** Digital volume correlation input parameters for displacement and strain measurements of copper-PLA sample.

Correlation parameter	Value
Subset Size	128 × 128 × 128
Mesh Spacing	16
Max Iteration	5
Overlap	50%
Convergence Criteria	0.25,0.5,0.0625
Cross-Correlation Threshold	0.0001

## Results and discussion

### Image segmentation

#### Greyscale distribution and particles

**Figure 10.**
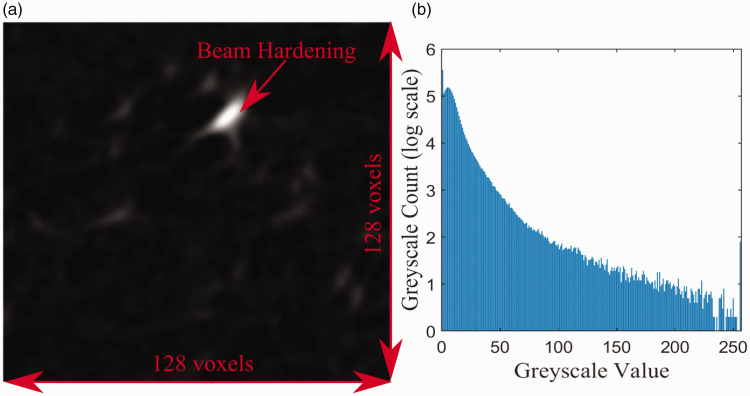
µ-CT data for 128 voxel correlation window size: (a) single µ-CT slice from correlation window, (b) resultant greyscale histogram for correlation window.

The quality of the obtained images was first assessed for the sample’s greyscale distribution. The ideal greyscale patterns utilized for DIC measurements exhibit a unimodal profile centering around mean greyscale values.^45–47^ Figure 10 shows a sample slice taken from the dataset and the entire subsets representative greyscale pattern. The greyscale histogram shows data with a peek at the low grayscale values representing black pixels within the image. Pan *et al.* has previously demonstrated the accuracy of a similar greyscale pattern for digital correlation measurement.^48^ Thus, while this does not display the typical greyscale distribution, it should still provide enough variation for correlation measurement to be obtained.

Before discussing the sample particle quantification, it is first necessary to discuss potential sources in error for the measurement. Observing Figure 10(a), one can quickly identify beam hardening and streaking within the image due to the metal particles. These features within the tomographs make a precise measurement of the copper particle’s volume challenging to resolve. Fortunately, particle quantity is a more meaningful indication of image performance for DVC measurement. Thus, the discussion here will be focused on the amount of the particles rather than their size. However, in regards to the DVC, it has been shown that beam hardening has little effect on the accuracy of displacement measurement.^49^ For these measurements’ particles smaller than 4.097 µm^3^, the volume of a resolved voxel was considered noise. Segmentation showed 1022 features with a volume above 4.097 µm^3^. This quantity is well above the minimum number required for an accurate correlation of 27 presented by Croom *et al.* and should provide an exact correlation.^50^

#### Cross-sectional analysis

**Figure 11.**
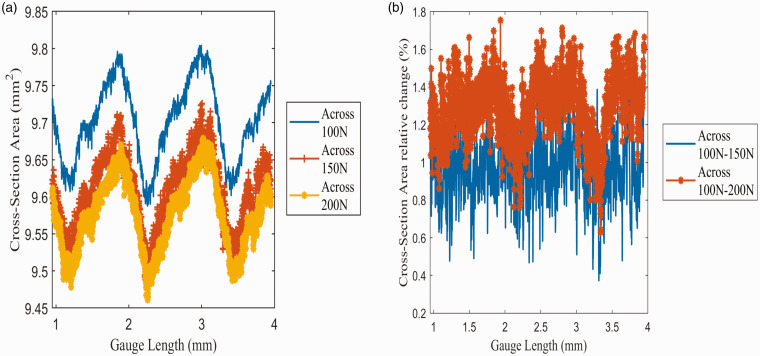
Variation of cross-section area through 3 mm section of the sample: (a) cross-section area variation through 3 mm gauge length of the sample, (b) percentage change of cross-section area compared to 100 N load.

The CT segmentation analysis of the cross-sectional area through a 3 mm section of each dataset is shown in Figure 11. Two clear trends can be seen regarding the cross-sectional area along the gauge length of the sample. The first trend seen is that each slice’s cross-section is shown to vary along the gauge length of material. Variation of the cross-sectional area is likely a result of manufacturing inconsistencies, which has led to specific layers forming larger than other layers. The standard deviation of the area along each slice is reported in Table 7 to quantify the cross-section area variation across the gauge length. It is shown here that variation in each segment lies in the range of ±0.0493 mm^2^ to ±0.0536 mm^2^. These tolerance ranges are well within the printer’s linear tolerance range of 0.1 mm to 0.3 mm. This cross-sectional area variation is reflected through the mean cross-sectional area of each sample: 9.7013 mm^2^, 9.6081mm^2^, and 9.5763 mm^2^ at the 100 N, 150 N, and 200 N loads, respectively. These areas are all slightly larger than the theoretical cross-sectional area of 9.42 mm^2^ obtained from the sample’s solid model.

**Table 7.** Statistical analysis of the cross-section area for different load conditions.

Sample load	Mean cross-section area (mm^2^)	Percent change from 100 N load	Standard deviation
100 N	9.7013	–	±0.0536
150 N	9.6081	0.9607	±0.0681
200 N	9.5763	1.2880	±0.0493

The second trend observed through the data is the reduction in the cross-sectional area due to the increased tensile load. As seen in Figure 11(b), the cross-sectional area has reduced between 0.4-1.8% of the preloaded condition. Furthermore, the mean area’s observation shows a resulting reduction in the area of 0.0932 mm^2^ and 0.125 mm^2^ or 0.9607% and 1.2880% from the preloaded state to 150 N and 200 N, respectively. This reduction in the cross-sectional area is reflected in a later section discussing the resolved DVC strain data. As a final note, as seen in Figure 10(a) the reduction of the cross-section is more significant between the 100 N to 150 N load than between the 150 N to 200 N. This is indicative of an increase in sample stiffness, which will be discussed in the porosity and air gap measurement section of this report.

#### Air gap analysis

Before making a quantitative assessment of the air gap volume, it is crucial to discuss the qualitative benefits of performing this CT segmentation procedure. As seen in Figure 8(d), segmenting out the sample air gaps, the print pattern can easily be identified. Within the sample, there are two significant sources of pores that can be recognized. The first source of porosity is between the sample’s shell layers and the infill of the material. The second is between the supporting infill raster of the material. As expected, these air gaps occur between the raster at an approximate +45°/−45° angle as specified by the print parameters. The orientation of these air gaps will play a significant role in assessing strain within the sample. As previously discussed by Tao *et al.*, features contained by air gaps support more of the tensile force.^51^

The total air gap volume is shown in Table 8. It is evident that even though the samples were sliced to be produced with an infill density of 100%, air gaps are still present between raster. As calculated through the CT segmentation procedure, the actual infill density lies between 99.2% to 99.5% as force is applied. As a result of the increasing tensile load samples, pores and air gaps begin to close. The changes in air gap size can be detected using the μ-CT analysis process. The closure of these air gaps is not linearly proportional to the applied load. The reason being is as these air gaps begin to close, there is a resultant increase in the young’s modulus of the sample.^17^ This increase in stiffness is likely why the decrease in cross-sectional area between the 100 N to 150 N is much more significant than between 150 N to 200 N.

**Table 8.** Air gap volume and actual sample infill density measured for a 3 mm section through µ-CT.

Applied force (N)	Air gap volume (mm^3^)	True infill (%)
100	0.025	99.2
150	0.020	99.4
200	0.017	99.5

### Digital Volume correlation of downsampled data

#### Sample displacement

**Figure 12.**
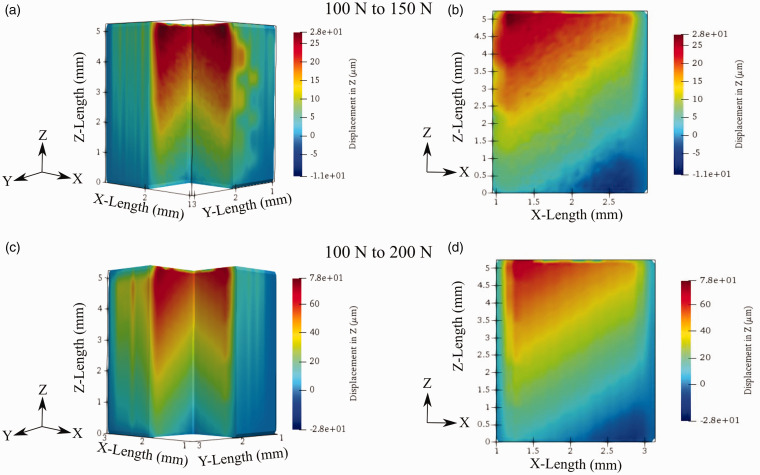
Displacement in direction (z-axis) of tensile load: (a) isometric view of displacement for 100–150 N test, (b) XZ plane displacement for 100–150 N test, (c) isometric view of displacement 100–200 N test, (d) XZ plane view of displacement for 100–200 N test.

While a wealth of information about the sample’s manufacturing and surface-level microstructure changes can be obtained strictly from sample segmentation, the DVC method was utilized for precise volumetric displacement and strain measurements. The longitudinal displacement fields are shown in Figure 12. Figure 12 provides two views of the resulting displacement fields between the preload condition of 100 N and the 150 N load and 200 N load. The longitudinal displacement is displayed with an isometric view and view of the XZ plane from the sample’s direct center. The isometric view aims to provide a 3 D view of displacement fields and highlight the displacement variation within the 3 D space. This view of experimental displacement variation within 3 D space is only possible to obtain via DVC. Simultaneously, the XZ plane’s displacement provides a detailed perspective of the total variation along the sample’s gauge length.

The gradient of displacements through the sample shows a typical development to ones obtained from DIC for +45°/−45° FFF samples under tensile loading.^52^ There is a displacement of 28 µm for the 100–150 N test and 71 µm for the 100–200 N test at the samples end closest to the load cell. This displacement slowly decreases toward the static end of the load cell. One would expect that at the stationary face, the displacement to be 0, but there is a small concentration of displacement present. The absence of an absolute static end is likely to present due to a lack of rigidity within the MTS, leading to a small region of longitudinal displacement.

While the displacement development is similar to ones obtained through DIC, a distinction can be made between the two based on the displacement pattern. Previous DIC results show longitudinal displacement in horizontal bands from the tensile face through the sample.^52^ DVC results from this study show the development of strain in angular segments throughout the piece. A probable cause for the evolution of displacement in this manner is due to incongruent loading between two the load pins attached to the sample’s end tabs. The development of this incongruent loading is possibly due to the nozzle starting position creating a stress concentration at the end of one side of the gauge length. This loading would result in a single end receiving slightly higher force than the other face, leading to angular displacement. Additionally, as previously mentioned, the MTS’s lack of rigidity could also contribute to unsymmetrical loading conditions. While this is a potential source of error, the purpose of this study is to prove the validity of DVC for displacement and strain analysis of FFF parts. Thus, the resulting strain field will be analyzed under the presumption that the developed force within the tensile coupon has been applied on a slight angle.

#### Sample strain analysis

**Figure 13.**
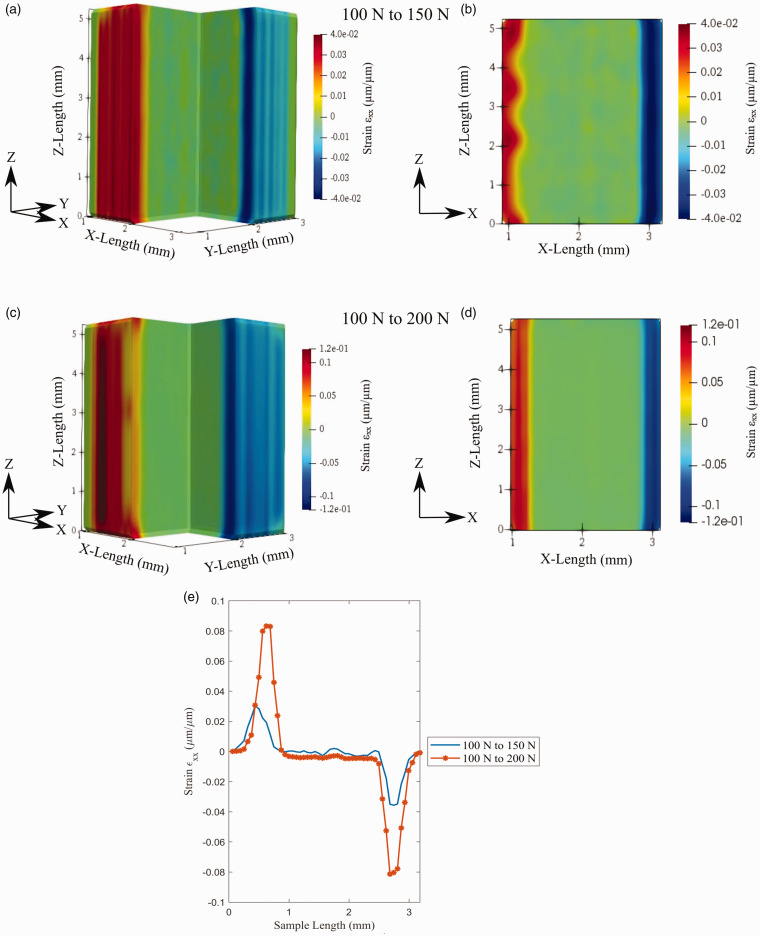
Volumetric strain $\epsilon_{xx}$ developed through the sample: (a) isometric view of 100 to 150 N test, (b) XZ plane view of 100 to 150 N test, (c) isometric view of 100 to 200 N test, (d) XZ plane view of 100 to 200 N test, (d) strain profile across the sample width.

Figure 13 displays an isometric view and XZ plane view of strain for the sample. Similarly to the displacement, this demonstrates a variation of the strain in 3 D space and provides a detailed outlook of strain in a single plane. Additionally, Figure 13 shows the 1 D development of strain along the x-axis about the samples’ center. Two loading states between the coupon are shown here, the first being between 100 N and 150 N and the second between 100 N and 200 N. It is seen that the strain develops mostly on the outer surface of the material, particularly within the shell of the sample. Comparing the isometric view to the XZ plane view, a positive strain is shown to develop on the shell’s leftward face and the area closest to the build plate. In contrast, the negative strain is realized in the sample’s rightward front and face furthest from the build plate. As expected, the strain between the 100 N and 200 N test is greater than the 100 to 150 N test indicating the overall cross-sectional contraction in the x-direction as the load increases.

**Figure 14.**
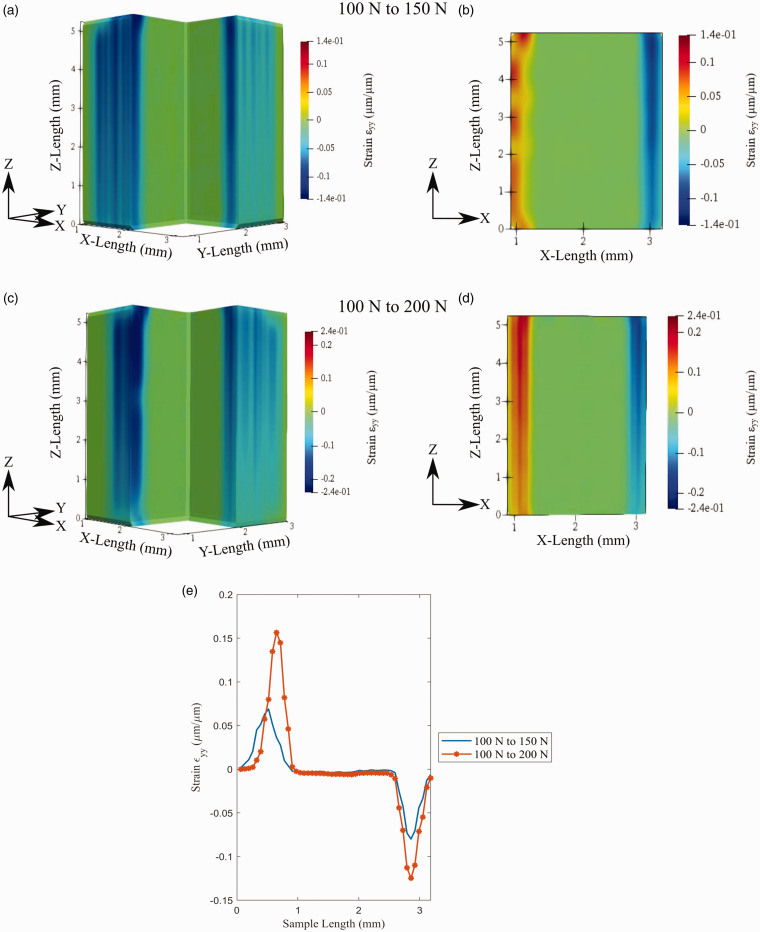
Volumetric $\epsilon_{yy}$ developed through the sample: (a) isometric view of 100 to 150 N test, (b) XZ plane view of 100 to 150 N test, (c) isometric view of 100 to 200 N test, (d) XZ plane view of 100 to 200 N test, (d) strain profile across the sample width.

Figure 14 demonstrates the isometric view and XZ plane for both the 100 N to 150 N load step and the 100 N to 200 N load step. As expected, the strain increased between the two different loading conditions. The transverse strain, ε_yy_, is shown to develop on the sample’s outer faces similar to that of the strain ε_xx_. This similarity is seen on the material’s shell as the leftward shell face is in positive strain, and the rightward face is experience strain in the opposite direction. For ε_yy,_ the strain on the sample face closest to the build plate is negative, while the face furthest from the build plate is positive.

Analyzing Figures 13 and 14 in conjunction provides details on the strain similarities between the transverse strain and the sample’s overall behavior. The development of strain in the transverse direction indicates the overall contraction of the coupon. It is notably shown that the material’s shell is reducing in size in the XY plane. This size reduction is reflected in the µ-CT tomographs, demonstrating an overall cross-sectional area across this sample plane. The development of strain within the shell of the sample is not entirely unprecedented. As previously mentioned, it has been reported that a higher concentration of force is supported within features that have surrounding air gaps.^51^ As observed in Figures 4 and, 8 large air gaps exist between the material’s shell and the infill. Thus, the material’s outer shell is likely bearing a more significant portion of stress, which is reflected through the development of strain.

Precise quantification of the transverse strain is plotted in Figures 13(e) and 14(e). The data shown within these graphics were collected by plotting the 1 D strain variation across the x-direction from the sample’s midpoint. The analysis of strain development of the material is reflected within these plots. Strain ε_xx_ and ε_yy_ are shown to peak approximately evenly on each face of the material. The 100 to 150 N dataset strain ε_xx_ is between 0.03 to −0.035, and ε_yy_ is between 0.069 to −0.080. The 100 to 200 N strain ε_xx_ is between 0.083 to −0.081, and ε_yy_ is between 0.157 to −0.125. The transverse strain peaks occur on the test samples’ outer faces, which are associated with the material shell. Measuring the mean width of the peaks for the 100 to 150 N and 100 to 200 N data, the strain peaks’ size is 0.78 mm and 0.75 mm. It can be seen that the size of these peaks relates closely to the slicer shell thickness shown in Table 1.

**Figure 15.**
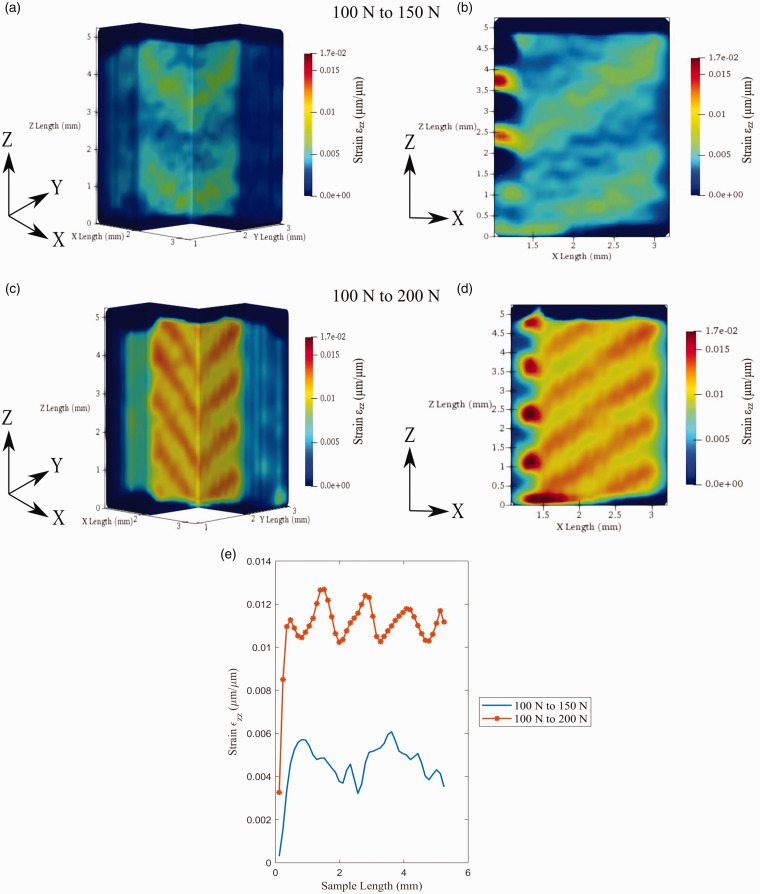
Volumetric strain ε_zz_ developed through the sample: (a) isometric view of 100 to 150 N test, (b) XZ plane view of 100 to 150 N test, (c) isometric view of 100 to 200 N test, (d) XZ plane view of 100 to 200 N test, (e) strain profile across the sample width.

The longitudinal strain (ε_zz_) uniquely does not develop on the sample’s shell but within the sample cross-section. This difference in strain development is indicative of the anisotropic and nonhomogeneous deformation of the sample. In Figure 15(a) and (b), the longitudinal strain develops within the infill at an angle. As the load is increased to 200 N, strain within the sample forms in periodic diagonal bands within the material’s bulk. These diagonal bands of strain appear to form at a 45° angle within the sample’s internal layers, which coincide directly with the raster infill pattern. This strain pattern has previously been seen for FFF printed parts within 2 D DIC studies but has not been previously shown volumetrically.^52^ Notably is the lack of strain forming on the −45° raster. This lack of strain development at a −45° angle relates to the previous discussion concerning the displacement fields. As previously mentioned, the sample is likely to be loaded on a slight angle. This uneven loading experienced by the sample results in the 45° bearing the majority of the load, while the −45° experience minimal loading.

Figure 15(c) displays the 1 D strain through the material’s gauge length about its center. The 100–150 N test is visibly less smooth, indication the beginning formation of strain within the sample. Subsequently, the 100–150 N presents a bimodal profile indicating only the initial strain formation within a few rasters. Observation of the 100–200 N test shows a much smoother profile, representing neatly formed bands of strains. An approximation of the strain band size was made from the strain data, resulting in mean size of 0.489 mm in width. This length relates directly to the rasters’ measure, which should have a theoretical size of 0.4 mm in width based on the nozzle size. The increase in raster size can be attributed to the DVC data’s discretization and the approximation made by not calculating the raster geometry directly perpendicular to the raster print direction. As a final comment, the longitudinal strain on the sample can be seen to be much smaller than the transverse strain shown figures (Figures 13 and 14) This is a direct result of the transverse strain developing on the sample’s shell, directly in line with the load cell.^51^ Thus, the material’s shell bearing a more substantial portion of the force and consequently experiences higher strain. Both the DVC transverse and longitudinal strain results can provide useful inputs for creating a complete FEA of FFF composites.^31–34^

## Conclusions

A uniaxial tensile test was conducted on a 3 D printed copper reinforced PLA tensile coupon within a µ-CT. According to ASTM-D648 14 Type V standards, the tensile coupon’s geometry was made with slight modifications to ensure compatibility with the utilized test stage. As a preliminary step toward the analysis displacement and strain analysis, the sample’s bulk structural response was measured through µ-CT image segmentation. The specimen was shown to have a bulk cross-sectional area larger than the solid model used to develop FFF but still within the printer tolerance. As the sample’s load increased, it was clear that the mean cross-sectional area was reduced due to elongation of the sample. However, the sample cross-section decrease was much more significant between the 100−150 N than 150–200 N indicating an increase in material stiffness as the sample was strained. The raster air gap was segmented from the sample and measured to support this statement. Two significant air gaps contributed to the sample’s porosity: 1) air gaps between the samples shell and infill, and 2) between the infill rasters. The formation of airgaps was aided in the analysis strain as features surrounded by air gaps are seen as regions that bear significantly more loading.^51^ The resulting volume of the air gaps showed a decrease in porosity as a tensile force increase, which has previously been shown to relate to a rise in sample stiffness.^17^

After segmenting the samples to determine DVC image compatibility, the resulting images were used as input into an open-source DVC software, FIDVC, to obtain the tensile coupon’s internal displacement and strain behaviour Lagrangian linear elastic strain formulation. Sample displacement had a similar gradient to typical tensile tests but was developed on an angle due to potential incongruent force application from the load cell. Thus all analysis for the strain fields was considered under the pretense of a slightly angular load condition. The resulting strain fields showed the effects of printing geometry on the development of strain within the sample. The axial strain on the part was developed on the 3 D printed structure’s surrounding faces, which relates to the overall reduction in the cross-sectional area seen from the image segmentation study. The development of this strain appears to form in 0.75–0.78 mm, which is associated with the shell thickness printing parameter

The longitudinal strain was shown to develop within the bulk of the material along the internal rasters uniquely. The development of strain along the rasters rather than the shell is due angularity of the rasters. The strain was observed to only develop on the +45° degree raster, but not the −45° raster, which relates to the force of load cell being skewed in +45° raster direction. Furthermore, the longitudinal strain developed within the sample was much less than the axial strain due to the shell’s alignment compared to the rasters. The sample shell in direct alignment with the load direction will bear a much higher degree of force than the rasters. DVC’s ability to capture the internal strain behavior provides a necessary first step towards a greater understanding of 3 D printed composites’ deformation behaviour. Future work on the effects of different print parameters and loads is essential for a complete understanding of these materials’ mechanical behaviour using the DVC measurement method. Additionally, these results can help build more intricate FEA models of FFF materials and allow for FFF parts to reach full potential in industrial applications.

## Supplemental Material

sj-pdf-1-jcm-10.1177_00219983211020500 - Supplemental material for Digital volume correlation analysis of polylactic acid based fused filament fabrication printed composites Click here for additional data file. Supplemental material, sj-pdf-1-jcm-10.1177_00219983211020500 for Digital volume correlation analysis of polylactic acid based fused filament fabrication printed composites by Cristofaro S Timpano and Garrett W Melenka in Journal of Composite Materials

sj-pdf-2-jcm-10.1177_00219983211020500 - Supplemental material for Digital volume correlation analysis of polylactic acid based fused filament fabrication printed composites Click here for additional data file. Supplemental material, sj-pdf-2-jcm-10.1177_00219983211020500 for Digital volume correlation analysis of polylactic acid based fused filament fabrication printed composites by Cristofaro S Timpano and Garrett W Melenka in Journal of Composite Materials
