# Supplementary material for: Digital volume correlation analysis of polylactic acid based fused filament fabrication printed composites
Source: J Compos Mater. 2021 May 27;55(25):3699–717. doi: 10.1177/00219983211020500 (PMC8551441; doi:10.1177/00219983211020500)
Supplement: sj-pdf-2-jcm-10.1177_00219983211020500 - Supplemental material for Digital volume correlation analysis of polylactic acid based fused filament fabrication printed composites [file sj-pdf-2-jcm-10.1177_00219983211020500.pdf]

Reviewer # 2 Comments

This paper presents various measurements of mechanical properties of FFF-printed composite by using micro-CT, DIC, DVC etc. Detailed measurement information is introduced. However, in consideration of the novelty, I do NOT recommend this work for publication in Journal of Composite Materials by its current form. The authors are strongly suggested to consider the following suggestions before final decision made by the editor.

The paper features various measurement techniques for 3D printed composites with particle reinforcement. However, these experimental testing methods are classical techniques in experimental mechanics. There is less work on the application of these approaches for the 3D printed composites with particles, but this cannot be considered as a main innovation. Instead, the authors need to outline more clearly what novel conclusions can be drawn from the conventional measurements or what are the challenges for the application of these classical techniques to the new structures.

For example, if the micro-CT or DIC has been applied to measure the mentioned 3D printed structures, the authors should clearly clarify the new contribution of this work. If not, the main difficulties or challenges during measurements, also with the solutions, should be highlighted. Only the detailed testing information is hard to achieve the novelty. This critical issue should be addressed appropriately before a reconsideration.

I have considered these comments and looked to address them in the start introduction of my paper

It is important to note that current method for the analysis of FFF and their composite are related to either traditional measurement techniques or DIC, but these come with many challenges and issues

For strain gauges and extensometers these are inherently discreet and surface level measurement techniques which means the full heterogenous deformation and anisotropic mechanical properties can be fully captures, while DIC alleviates the discreet nature of strain gauges, and extensometers, it itself also a surface level technique

The print parameter of FFF parts have a major effect on the microstructure which effect the overall mechanical properties of FFF parts and their composites, thus a new technique was needed to directly relate the microstructure of composite during loading to deformation behavior which is the fundamental challenge we are trying to meet here through μ-CT and DVC

Not only will this provide us with a better understanding the deformation behavior of FFF composite, but will provide a fundamental starting point towards future works that aim for a more complete understanding of print parameters effect on the microstructure and mechanical properties and deformation behavior of FFF parts.

In addressing this comment, I have added the following sentences:

“This is due to strain gauges and extensometer being inherently a discreet surface level measurement system. Thus, the anisotropic material properties and heterogenous deformation field can’t be captured. Additionally, the build parameter of FFF parts intrinsically effect the mechanical properties, by way of altering the microstructure of the material, which can’t be captured by of a strain gauge which is macroscopic measurement technique (7).”

“Thus, it is difficult to capture and relate strain results throughout the entire 3D microstructure, which is crucial for materials which are highly influenced by there microstructure, via the DIC technique.”

“The major challenge that this work is looking to address is to capture full 3D deformation and strain measurement as they relate to the microstructure of FFF materials. As build parameters directly influence the microstructure of FFF materials and thus their mechanical properties, current methods such as strain gauges, extensometers, and DIC are incapable of capturing this interaction. μ-CT and DVC provide a method for capturing the microstructure of FFF parts, and its deformation during loading which can be directly converted to strain results. This paper provides a fundamental first step towards capturing the deformation behaviour of FFF parts, which can be built upon in future works that aim to provide a more complete analysis of build parameter effects on the deformation mechanics of 3D printed parts and their composites.”
