# Supplementary material for: Digital volume correlation analysis of polylactic acid based fused filament fabrication printed composites
Source: J Compos Mater. 2021 May 27;55(25):3699–717. doi: 10.1177/00219983211020500 (PMC8551441; doi:10.1177/00219983211020500)
Supplement: sj-pdf-1-jcm-10.1177_00219983211020500 - Supplemental material for Digital volume correlation analysis of polylactic acid based fused filament fabrication printed composites [file sj-pdf-1-jcm-10.1177_00219983211020500.pdf]

Response to Reviewer #1 Comments

1. In the introduction section, the authors should explain why they believe that conventional strain testing methods (strain gauges and extensometers) are unreliable for their material system.

Conventional strain measurement methods are not suitable as they are inherently discreet surface level measurement

When dealing FFF composites with it is important to note that the mechanical properties are anisotropic and heterogenous in nature, thus a true full field view of strain can't be obtained from strain gauges.

Furthermore, the mechanical properties are affected by build parameters which alter the microstructure and intern effect the mechanical properties.

These properties can't be capture with traditional strain gauges or DIC, thus why DVC was utilized to capture the strain results more accurately

The following sentences were added to highlight this point:

"This is due to strain gauges and extensometer being inherently a discreet surface level measurement system. Thus, the anisotropic material properties and heterogenous deformation field can't be captured. Additionally, the build parameter of FFF parts intrinsically effect the mechanical properties, by way of altering the microstructure of the material, which can't be captured by of a strain gauge which is macroscopic measurement technique (7)."

"Thus, it is difficult to capture and relate strain results throughout the entire 3D microstructure, which is crucial for materials which are highly influenced by there microstructure, via the DIC technique."

2. In the methodology section, the authors should provide details about the gripping methods in their tensile experiment (grip type, pressure,...etc).

The coupon was held in place by on both ends by 2 pairs of base plates, one pair of base plates had a smooth hole and the other a threaded hole

Pressure was applied by passing the screw through the smooth hole, past the sample and into the threaded hole where it was tighten to apply pressure to the sample

I have added this sentence to the paragraph to aid in understanding the design of the grips:

"The tensile coupon is connected to the MTS with top and bottom clamps. Pressure is applied to the sample through the clamps through by a pair of screws which pass through the test coupon"

3. In section 2.5, what is the rationale for choosing the Lagrangian strain formulation over the other strain calculation formulas?

The Lagrangian linear elastic strain was chosen due to the brittle nature of FFF material and low loads

Brittle materials remain in their linear elastic region until fracture, thus since the loads applied did not fracture the material this strain formulation was utilized

This sentence has been added to highlight this "The Lagrangian linear elastic strain formulation was chosen due to the brittle nature of FFF and low load applied resulting in the sample remaining in this strain region prior to fracture (40)."

4. Figure 10(a), add labels, circles/arrows to enhance the visibility of beam hardening.

I have added an arrow highlighting the beam hardening to enhance the visibility

5. It would be quite useful if the authors add a separate discussion about the influence of the process parameters (build orientation, infill pattern, temperature, feed rate,..etc) on the tensile behavior.

We have begun to address this in point 1 when discussing why strain gauges, and extensometers are not appropriate

However, we want to further highlight this point in discussing the major challenge that this paper is trying to address, which is development of a method for analyzing the strain and material loading behaviour of a FFF that directly relates the microstructure to the strain data

This paper is focused on utilizing this technique as a fundamental starting point showing the potential for DVC in obtaining 3D strain result that or intrinsically linked to the microstructure

In doing so future research can be done for the analysis of process parameter effects on strain directly through the microstructure of the material

In addition to the sentences added in point 1, I have added the following paragraph

"The major challenge that this work is looking to address is to capture full 3D deformation and strain measurement as they relate to the microstructure of FFF materials. As build parameters directly influence the microstructure of FFF materials and thus their mechanical properties, current methods such as strain gauges, extensometers, and DIC are incapable of capturing this interaction. μ-CT and DVC provide a method for capturing the microstructure of FFF parts, and its deformation during loading which can be directly converted to strain results. This paper provides a fundamental first step towards capturing the deformation behaviour of FFF parts, which can be built upon in future works that aim to provide a more complete analysis of build parameter effects on the deformation mechanics of 3D printed parts and their composites."
